# Horse vision and obstacle visibility in horseracing

**DOI:** 10.1016/j.applanim.2019.104882

**Published:** 2020-01

**Authors:** Sarah Catherine Paul, Martin Stevens

**Affiliations:** Centre for Ecology & Conservation, University of Exeter, Penryn Campus, Penryn, TR10 9FE, UK

**Keywords:** Horse, Equine, Vision, Animal welfare, Safety, Racing, Behaviour, Sports

## Abstract

Visual information is key to how many animals interact with their environment, and much research has investigated how animals respond to colour and brightness information in the natural world. Understanding the visibility of features in anthropogenic environments, and how animals respond to these, is also important, not least for the welfare and safety of animals and the humans they co-exist with, but has received comparatively less attention. One area where this is particularly pertinent is animal sports such as horseracing. Here there is a need to understand how horses see and respond to obstacles, predominantly fences and hurdles, as this has implications for horse and rider safety, however obstacle appearance is currently designed to human perception. Using models of horse colour and luminance (perceived lightness) vision, we analysed the contrast of traditional orange markers currently used on fences from 11 UK racecourses, and compared this to potential alternative colours, while also investigating the effect of light and weather conditions on contrast. We found that for horses, orange has poor visibility and contrast against most surroundings. In comparison, yellow, blue, and white are more conspicuous, with the degree of relative contrast varying with vegetation or background type. Results were mostly consistent under different weather conditions and time of day, except for comparisons with the foreground turf in shade. We then tested the jump responses of racehorses to fences with orange, fluorescent yellow, bright blue, or white takeoff boards and midrails. Fence colour influenced both the angle of the jump and the distances jumped. Bright blue produced a larger angle of takeoff, and jumps over fluorescent yellow fences had shorter landing distances compared to orange, with bright blue fences driving a similar but non-significant trend. White was the only colour that influenced takeoff distances, with horses jumping over white fences having a larger takeoff distance. Overall, our results show that current obstacle coloration does not maximise contrast for horse vision, and that alternative colours may improve visibility and alter behavioural responses, with the ultimate goal of improving safety and welfare.

## Highlights

• Using white, fluorescent yellow, or bright blue for fence markers would provide higher contrast for horses than the current orange markers.
• Contrast is maximised across light and weather conditions by highly luminant whites or blues at the base of the fence (take-off board).
• Fluorescent yellow has the greatest contrast against the main fence body (i.e. when used for midrail colour) across different light and weather conditions.
• Horses jumped differently over white, fluorescent yellow, and bright blue marked fences compared to orange marked fences.

## 1 Introduction

Visual information is key to guiding appropriate behaviour in many species, including avoiding threats and in navigation and orientation (Cronin et al., 2014). Owing to a variety of factors, including ecology and life-history, visual abilities and characteristics vary enormously among animals, meaning that many species see the world very differently (Stevens, 2013). This is most readily apparent with colour vision, which can vary from those species that lack the ability to discriminate colour (monchromats), to those that are di-, tri-, tetrachromatic, and even potentially beyond (Hadfield et al., 2007). This becomes crucial when considering the way animals, both wild and domestic, navigate environments designed by humans, and therefore from a human visual perspective. One such example is in animal sports, where the sport and associated traditions commonly pre-date a broad knowledge and understanding of animal vision (DeMello, 2012). Consequently, due to differences between human vision and that of animals used in competitions, important features of the sporting, training, and housing environments may not be well-designed for visibility to the focal animal itself. Recent advances in the animal visual sciences mean that we now have the opportunity to re-assess these environments using approaches designed to quantify and predict how animals see and respond to visual information (Kelber et al., 2003a; Kelber and Osorio, 2010; Gawryszewski, 2018).

The above considerations are particularly relevant for horse sports, which represent some of the most watched spectator sports worldwide (Albrecht et al., 2012), and frequently attract attention regarding ethics and welfare (Graham and McManus, 2016; Markwell et al., 2017). In most horse sports, particularly in those disciplines that involve jumps, visual information is crucial, enabling horses (and their riders) to safely navigate obstacles like fences and hurdles. Often, a key aim is to balance a challenging set of conditions that will test rider and horse while maintaining safety standards. In jump racing (also known as National Hunt Racing in the UK), how horses see and respond to fences and hurdles is likely to influence the probability of falls and related problems. In the UK, the governing body of horseracing, the British Horseracing Authority (BHA), report that an average of 176 horses have died in the UK each year over the past 5 years as a result of racing (“BHA Equine Injuries and Fatalities data for, 2017”). Although this data is spread out across all types of competitive equine track racing, it is well established that the majority of fatalities occur in jump racing, often due to incidents at jumps (Pinchbeck et al., 2004, 2002; Williams et al., 2001). It is clear, therefore, that a major consideration in the welfare and safety of horses and jockeys in jump racing is the need to reduce the number of falls and injuries at fences and hurdles.

**Fig. 1:**
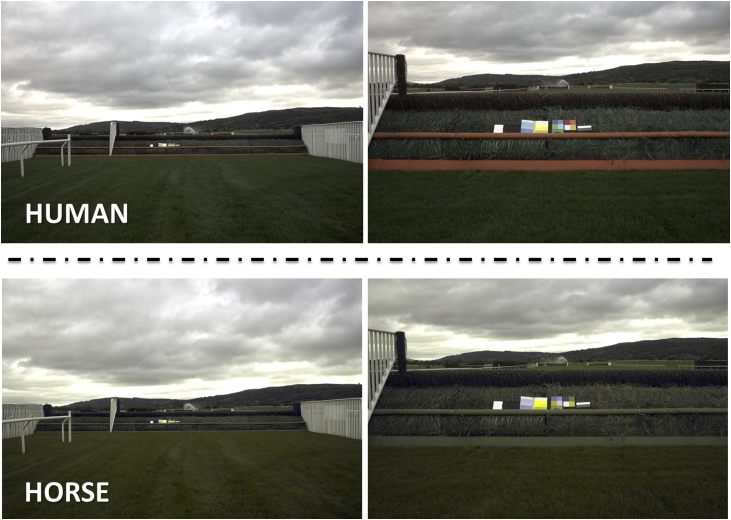
A fence at Cheltenham racecourse on an overcast day to human and predicted horse vision. Images illustrate the much higher contrast of white, fluorescent yellow, and blue (in the colour boards) to the fence and its surroundings than the orange takeoff board and midrail.

The contrast of an obstacle against its surroundings is important in enabling the determination of obstacle presence, size, and the distance between the viewer and the obstacle (Bruce et al., 2003). Currently, the visibility markers that help demarcate the presence of fences (the takeoff board and midrail) and hurdles in jump racing are orange. This makes them conspicuous to humans with ‘normal’ colour vision; i.e. trichromats, who see colour based on three cone types sensitive to relatively short- (‘blue’), medium- (‘green’), and longwave (‘red’) parts of the spectrum (Bowmaker and Dartnall, 1980). In comparison, horses have dichromatic colour vision, with two cone types, sensitive to short (428 nm peak) and medium wavelengths (539 nm peak) (Carroll et al., 2001). This means that they have reduced colour vision compared to humans, seeing colours along a continuous range from blue to yellow (Macuda and Timney, 1999; Roth et al., 2008, 2007; Smith and Goldman, 1999), and therefore cannot distinguish between many of the colours that humans see as red, orange, and green, unless they also differ in brightness (Murphy et al., 2009). The orange fence markers used in racing may therefore increase the visibility of fences against the background to a far lesser extent for horses than for humans, and this may be exacerbated under certain light conditions, weather, and with variation in the visual appearance of different types of vegetation (Fig. 1.). However a thorough investigation into the conspicuousness of current markers to horses is lacking.

Our key aims here were twofold: first, to compare the predicted visibility of current fences and hurdles (both internal and external contrast) with alternative, and potentially more conspicuous, colours across a range of different light conditions (weather and time of day). Second, as it is important not only to determine how horses might see specific colours, but also how they respond to these colours in their environment (Kelber and Osorio, 2010; Stachurska et al., 2010, 2002) we assess the behavioural responses of racehorses to alterations in a select number of fence colours, guided by the first analysis. We analysed the visibility of fences and hurdles from 11 racecourses used in jump racing to low-level (photoreceptor) colour vision models of horse vision, using image analysis techniques (for full description see: Stevens et al., 2007; Troscianko and Stevens, 2015). We analysed the predicted visibility of the current fence and hurdle colours (orange), and a range of alternative colours, against the fence, fence foreground, and fence background. We then undertook behavioural trials with racehorses in a training setting, in order to compare the jumping response of horses to the traditional orange coloured fence markers, versus fence markers with three colours identified by the visual modelling analysis as being more contrasting to horses. Based on a knowledge of horse colour vision, we predicted that the commonly-used orange colour of fences and hurdles would be hard to see for horses under a variety of conditions, and that three other colours (white, fluorescent yellow, and blue) would be more contrasting against fences/hurdles and their surroundings. Furthermore we predicted that more visible alternative colours, when used to colour fences in the behavioural trials would influence horse jumping behaviour.

## 2 Methods

### 2.1 Quantifying obstacle visibility to horse vision

Eleven different racecourses around the UK (Aintree (Mildmay & Grand National), Chepstow, Cheltenham, Exeter, Hereford, Ludlow, Newton Abbott, Stratford, Taunton, Wincanton, Worcester) were visited to assess fence appearance to horse vision (February 2017-February 2018). Furthermore, during this period Exeter was visited four times, Chepstow, Ludlow, Newton-Abbott, and Taunton were visited three times and Wincanton twice in order to investigate the effects of light conditions and weather. Digital images of 131 fences and hurdles were taken across all courses and converted to horse vision (see below). This enabled us to analyse the level of visual contrast (visibility) for colour and luminance of different fence and hurdle features. Specifically, we calculated three key aspects of visibility: i) fence takeoff board against the foreground (e.g. turf in front of the obstacle), ii) top part of the fence (e.g. brush material) or hurdle against the visual background (e.g. trees or sky), and iii) the contrast of the fence midrail with the surrounding internal areas of the fence material. In addition, we conducted the same comparisons, but substituting a range of different colours and materials, ranging from red and fluorescent yellow to blue (see below for full details), in order to test whether alternative colours would be more conspicuous to horses than the colours and materials currently used on fences and hurdles in UK racing. In addition, we investigated the effect of light conditions (weather and time of day) on the visibility of both traditional colours used in racing (orange), as well as the most contrasting colours identified in our initial analyses. This allowed us to establish whether certain colours may be more contrasting under different light conditions.

Individual fences were photographed using a Sony A7 digital camera fitted with a Sony 28–70 mm F3.5–5.6 FE OSS stock lens with two diffuse PTFE reflectance standards (20 × 20 cm) of known reflectance (white: 93.1% and black: 4.49%) and a pair of colour boards in each image (Fig. 1.). Fences were photographed at a distance approximating four gallop strides out plus takeoff (∼32 m). The colour boards used were one of three different types and were designed to enable us to investigate the visibility of a range of colours to horses under the same conditions as the fences/hurdles and the results were used to inform the choice of colours used for the behavioural trials. The first two boards consisted of rectangles of yellow (Y), orange (O), red (R), dark green (DG), medium green (MG), light green (LG), dark blue (DB), medium blue (MB), light blue (LB), white (W), and black (B) ethylene-vinyl acetate (EVA), used for its low (<5%) reflectance. One board was matt and the other had a covering of glossy Fablon® Vinyl (shiny). The comparative contrast of these coloured rectangles (see below for methods) was used to identify colours that would be the most conspicuous under horse vision (white, yellow, and blue). A third board was then used to discern the most appropriate shade and/or material of these three colours (i.e. the most contrasting) for use in the behavioural trials. This third board consisted of rectangles of white EVA (W), white paint (WP), light blue paint (LBP), light blue EVA (LB), med blue tape (MBT), white paint (WP), fluorescent yellow card (FLC), fluorescent yellow tape (FYT), and yellow paint (YP) with half of each rectangle covered in glossy Fablon® Vinyl (shiny). The key aim was to investigate a wide range of colours and shades. The weather and light conditions were split into eight different classifications, by the time of day (daytime or evening) and by the weather conditions (sunny, sunny with cloud cover, overcast, and shade). Photographs were taken during the day or during the evening (<3 h before sunset), as racing occurs predominantly in the afternoon and early evening and evening light has a different spectral quality (Endler, 1993) and low lying sun is often suggested to cause problems at racecourses, although predominantly due to issues with glare Weather was classified as sunny (<10% cloud cover), sunny with cloud cover (bright conditions with 20–60% cloud cover), and overcast (grey with 80–100% cloud cover), with an additional category added for those fences on sunny days that were in shade due to the direction of the sunlight.

Digital image analysis and vision modelling were used to quantify values for each fence or colour and contrast with the background, as per the three comparisons above (Kelber et al., 2003b; Osorio and Vorobyev, 2005; Troscianko and Stevens, 2015). Images were taken in RAW format with manual camera settings. To correct for the non-linear response of the camera to light levels (radiance), and for any variation in light levels between photos, each image was linearized with respect to light intensity and equalized with respect to the standards (Stevens et al., 2007). This was carried out using the programme ImageJ 1.49 t and the Multispectral Image Calibration and Analysis Toolbox plugin (Troscianko and Stevens, 2015). Next, using a widely implemented image transformation approach (Pike et al., 2010; Stevens et al., 2007; Stevens and Cuthill, 2006; Troscianko and Stevens, 2015), images were mapped to the predicted responses of horse visual systems, using horse spectral sensitivity (Carroll et al., 2001). This mapping technique is highly accurate compared to modelling photon catch data with reflectance spectra (Troscianko and Stevens, 2015). This resulted in predicted cone catch data for the horse shortwave (SW) and longwave (LW) receptors.

Key areas of each fence and the foreground and background were then selected and measured. In order to predict the degree to which fence/hurdle colours, and those on the colour boards, were distinguishable from the foreground, background, and internal fence material, we used a commonly-implemented log version model of visual discrimination that takes into account variation between receivers with different visual systems and is based on the concept that receptor noise limits visual discrimination (Osorio and Vorobyev, 1998). The output is given as ‘Just Noticeable Differences’ (JNDs), where values under 1 equate to low, 1–3 poor, and >3 increasingly good contrast between the respective fence components. Colour and luminance JNDs were calculated using the longwave and shortwave photoreceptor (cone) data and Weber fractions of 0.05, using values for LW to SW cone abundance (40:5 - based on average SW cone abundance across entire retina; 26).

### 2.2 Behavioural responses to different fence colours

For the experiment testing behavioural responses to different fence colours, we used horses trained at Richard Phillips Racing. Work was conducted under approval from the University of Exeter Biosciences Ethics Committee (application 2018/2100). The jump trials were carried out Adlestrop Stables (Adlestrop, Moreton-in-Marsh, Gloucestershire, GL56 0 YN) by two professional jump jockeys.

**Fig. 2:**
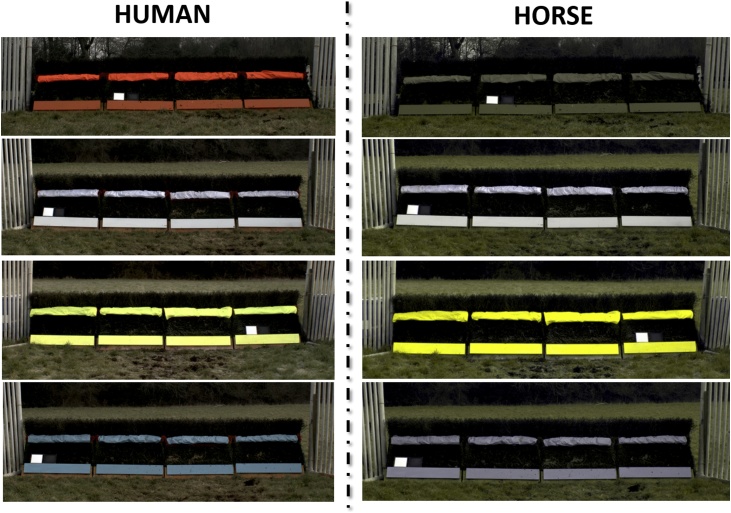
Photos of all four colours of experimental fence used in the behavioural trials, on the right in human (jockey) vision and on the left in predicted horse vision. Fence colours are from top to bottom orange (traditional), white, fluorescent yellow, and bright blue.

A total of 14 horses were trialled over a pair of jumps that differed only in the colour of the takeoff board and midrail. Each horse was jumped over a pair of fences three times. One fence in each pair had a classic orange takeoff board and guard rail, whereas the takeoff board and guard rail on the other fence were either white, fluorescent yellow, or bright blue (Fig. 2). To account for order effects, the alternative fence colour (white, fluorescent yellow, or bright blue) was used on both the first and second fences, leading to a total of six different fence combinations (Fence 1- Fence 2): orange-white (n = 10), orange-fluorescent yellow (n = 5), orange- bright blue (n = 9), white-orange (n = 6), fluorescent yellow-orange (n = 8), bright blue-orange (n = 7). Takeoff boards consisted of a wooden board (0.11 m by 4.6 m), painted in either orange, white, fluorescent yellow, or bright blue, and fixed securely to the base of the fence. The guard rail was coloured using PU coated Nylon Ripstop fabric (0.14 m by 4.6 m) in either orange, white, fluorescent yellow, or bright blue, and securely fastened to the middle of each fence. The number of horses that jumped each combination and the jockey that rode them varied between treatments, due to racing schedule constraints.

All trials were filmed using an SJCAM (720p 1280*720 60fps) set at approximately 9 m perpendicular to each fence. Still frames of each jumping effort were then extracted from the footage (Wejer et al., 2013) and corrected for lens distortion (Lens Analyzer, Chaos Utility - Version 1.10). The undistorted images were then imported into Image J, and eleven different jumping parameters (Table 1) were measured, using the first three bars of each fence to establish the scale. The eleven different jumping parameters measured are frequently used to assess jumping performance across a range of equine sports (de Godoi et al., 2016d, 2014; Lewczuk et al., 2006; Lewczuk and Ducro, 2012).

**Table 1:** A description of each of the eleven different jumping parameters measured in the behavioural trial with an example image to illustrate how each measurement was made on video stills. The lines in each image correspond to the line numbers given in parenthesis in the description box.

Measurement	Description	Image
**Takeoff distance 1**	Distance from Front Leading Limb and fence base on anterior side.	
**Takeoff distance 2**	Distance from Front Trailing Limb and fence base on anterior side.	
**Takeoff distance 3**	Distance from Hind Leading Limb and fence base on anterior side.	
**Takeoff distance 4**	Distance from Hind Trailing Limb and fence base on anterior side.	
**Angle of takeoff**	Measured as two lines, emanating from the hind quarters of the horse (between the sacral vertebrate and the croup). The first line runs from the croup towards the forelimbs (parallel to the ground), and the second runs along the dorsal side of the horse towards the withers.	
**Height of wither at jumping**	The maximum height of the withers (point between the scapula on the dorsal side of the horse) during the jump from the top of the obstacle (i.e. fence).	
**Angle of Bascule**	Measured from the hind quarters to the withers and then the withers to the ears, it is the lower angle between these two lines and represents the jump mid-point.	
**Landing distance 1**	Distance from Front Leading Limb and back base of fence	
**Landing distance 2**	Distance from Front Trailing Limb and back base of fence	
**Landing distance 3**	Distance from Hind Leading Limb and back base of fence	
**Landing distance 4**	Distance from Hind Trailing Limb and back base of fence	
**Total jump distance**	Distance between the hind leading limb at takeoff and the front trailing limb at landing.	

### 2.3 Statistics

Data were analysed using R version 3.4.3 (R Core Team, 2017). Alpha level was set at 0.05 for all tests, analyses carried out using liner mixed effects models (package = lme4; see below for full description of each model), model residuals were checked for normality and variance homogeneity, and stepwise backwards deletion using Chi-square likelihood ratio tests (package:MASS) was employed to reach the minimum adequate model (Crawley, 2012). For the analysis of obstacle and colour visibility at racecourses under different light conditions, variation in contrast (colour and luminance JNDs) was tested using a linear mixed effects model where: either Colour or Luminance JND was the response variable; and the fixed effects were the fence or colour board component identity (e.g. midrail, yellow, blue, or white), the light conditions (a combination of the weather and time of day: overcast in the day (Overcast_Daytime), overcast in the evening (Overcast_Evening), shade during the daytime (Shade_Daytime), shade during the evening (Shade_Evening), sunny with cloud cover in the daytime (Sunny_CloudCover_Daytime), sunny with cloud cover in the evening (Sunny_CloudCover_Evening), sunny in the daytime (Sunny_Daytime), and sunny in the evening (Sunny_Evening)), and their interaction (Fence/Colour* Light Conditions). Course (Course_ID, e.g. Aintree_National) and fence identity (Fence_ID, e.g. Aintree_National_Fence_1) were included as random effects, with fence nested within course. Colours investigated were white, yellow, and blue as these were already identified as having significantly higher contrast to the fence or surrounding environment than the current fence colours used (see results section). To increase the power of the analysis, and because not all shades were photographed under all lighting conditions, different material types and colour shades were pooled for the weather analysis (e.g. white = white EVA and white paint). The analysis was carried out for the luminance and colour JND differences for each of the three fence edge comparisons; foreground vs. takeoff board (colour JND and luminance JND), fence vs. midrail (sqrt colour JND and untransformed luminance JND), and fence edge vs. fence background (sqrt colour JND and sqrt luminance JND), with transformations being applied to response variables where appropriate to improve model fit. Specific post-hoc comparisons were made between the JND values for each of the test colours (white, yellow, and blue) and each of the fence components (takeoff board, midrail, and the edge of the top of the fence i.e. fence edge) within each of the eight different light conditions (package = multcomp, Hothorn et al., 2008).

The effect of fence colour on each of the different jumping parameters measured was tested using a linear mixed effects model, where each jumping parameter (e.g. total jump distance) was a response variable; fence colour, fence sequence (the first or second fence in the pair of fences), and jump number (whether it was the 1^st^, 2^nd^, 3^rd^, or in rare cases 4^th^ time a horse had jumped the pair of fences) were fixed variables; and horse ID and trial day were the crossed random effects. The random effect of horse ID was included to account for the use of the same horses over multiple trials, and trial day to control for the variation between trials in jockey, weather conditions and the order in which the fences were jumped (i.e. Fence 1 = Orange and Fence 2= Test Colour (white/fluorescent yellow/bright blue)). Where colour was identified as having a significant effect on any of the jumping parameters measured specific post-hoc comparisons (package = multcomp, Hothorn et al., 2008) were made to assess differences in the parameter of interest (e.g. total jump distance) between jumps made over orange fences and those made over fences of each of the three test colours (white, fluorescent yellow, and bright blue).

## 3 Results

### 3.1 Obstacle visibility to horse vision

The visibility of fences is strongly affected by colour type (e.g. orange or blue) and luminance (e.g. light blue or dark blue). Current colours and materials used for the takeoff boards, midrails, and top edge of fences (orange paint, orange waterproof material, and natural vegetation) offer variable and frequently low visibility to horses, whereas other colours such as blue, yellow, and white offer much higher visibility (Table 2, Table 3, Table 4; Fig. 1 & 2 ).

**Table 2:** Foreground vs Fence - Colour and Luminance visibility data (JNDs) for fence components and alternative potential colours against the foreground turf. N = the sample size of images/comparisons made, SE is the standard error (a measure of variation in the measurements across samples). JNDs are discrimination values from the horse colour and luminance (perceived lightness) models. These reveal how visible an object is predicted to be against a given background. Higher JND values indicate a colour is more visible. (For interpretation of the references to colour in this table, the reader is referred to the web version of this article).

**Table 3:** Midrail vs Fence (hedge/brush) - Colour and Luminance JNDs for midrail and alternative potential colours against the rest of the fence. N = the sample size of images/comparisons made, se is the standard error (a measure of variation in the measurements across samples). JNDs are discrimination values from the horse colour and luminance (perceived lightness) models. These reveal how visible an object is predicted to be against a given background. Higher JND values indicate a colour is more visible. (For interpretation of the references to colour in this table, the reader is referred to the web version of this article).

**Table 4:** Fence/Hurdle vs Background - Colour and Luminance JNDs for fence components and alternative potential colours against background behind the fence or hurdle. N = the sample size of images/comparisons made, se is the standard error (a measure of variation in the measurements across samples). JNDs are discrimination values from the horse colour and luminance (perceived lightness) models. These reveal how visible an object is predicted to be against a given background. Higher JND values indicate a colour is more visible. (For interpretation of the references to colour in this table, the reader is referred to the web version of this article).

#### 3.1.1 Predicted visibility of current fence/hurdle colours

The colours currently used on fences and hurdles offer low predicted visibility to horses. In many cases, there is low predicted visual contrast between the bottom of the fence and its foreground, the midrail and adjacent fence components, and the top of the fence and its background. Woody and orange coloured edges in particular have low predicted visibility, particularly in terms of chromatic contrast against the foreground (Table 2, Table 3, Table 4), and are substantially less visible than some of the potential alternative colours we tested. The type of material used (e.g. gloss versus matt) also plays a role in the predicted visibility – with matt offering better contrast than gloss for the majority of colours tested (Table 2, Table 3, Table 4).

#### 3.1.2 Predicted visibility of potential alternative obstacle colours

The use of white, yellow, or blue is predicted to improve the visibility of the takeoff board, midrail, and top of the fence to horses (Table 2, Table 3, Table 4). The exact shade, texture, and/or brightness properties of the white, yellow, or blue used influences the conspicuousness of these colours. Light blues provide higher luminance contrast than darker blues (Table 2, Table 3, Table 4) and matt fluorescent yellow consistently has the highest colour and luminance contrast of all the colours tested. Consequently in light of these results white, fluorescent yellow, and light blue were compared to the classic orange for the behavioural response experiments in this study.

#### 3.1.3 Role of weather conditions

**Fig. 3:**
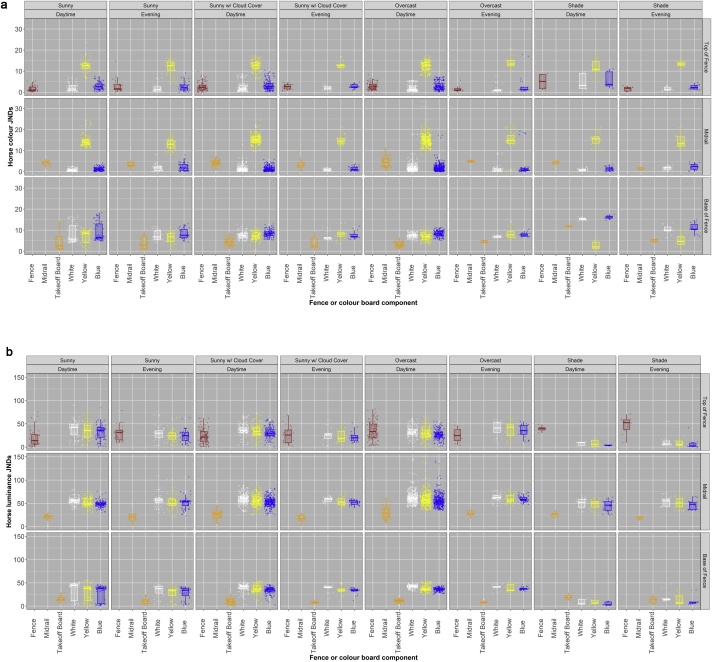
a) Colour JNDs and b) Luminance JNDs of fence components (fence/midrail/take-off board) and alternative potential colours (white, yellow, and blue) against either the fence background/fence/fence foreground (turf), for horse vision under different light conditions (weather and time of day). JNDs are discrimination values from the horse colour and luminance (perceived lightness) models. These reveal how visible an object is predicted to be against a given background. Higher JND values indicate a colour is more visible.

The predicted visibility of orange, white, yellows, and blues is affected by light conditions, vegetation, weather, and shadows (Figures 3a & b). Light conditions can substantially influence the contrast of both the chromatic (takeoff board/colour board * light conditions: X^2^_1, 21_ = 216.01, P < 0.001) and achromatic (takeoff board/colour board * light conditions: X^2^_1, 21_ = 186.90, P < 0.001**)** components of these colours against the fence foreground (turf). **White,** yellow, and blue all have higher contrast than the standard **orange** under sunny and overcast conditions. However, in evening shade, yellow has reduced contrast to **orange** whereas **white** and blue remain much more highly contrasting (Fig. 3a & b; Supplementary Material 1). This pattern does not occur for luminance, however, where shade reduces the luminance contrast of all three test colours **(white,** yellow and blue) equally, resulting in similar luminance contrast of the three test colours to the **orange** takeoff board, which has low luminance contrast across all light conditions. Yellow has the greatest colour contrast to internal fence components across all light conditions (midrail/colour board * light conditions; X^2^_1, 21_ =127.23, P < 0.001; Fig. 3a & b; Supplementary Material 1). Yellow, white, and blue all have similar levels of luminance contrast to the fence across all light conditions, as well as all having greater luminance contrast than the traditional orange midrail (non-significant interaction between midrail/colour board and light conditions; X^2^_1, 21_ =*28.29,* P *= 0.13*; Figures 3a & b). Light conditions significantly influenced both the colour and luminance contrast of the fence or colour board colour against the background [significant fence/colour board interaction for colour JNDs (X^2^_1, 21_ **=**33.77, P*=* 0.032) and luminance JNDs (X^2^_1, 21_ **=**227.20, P < 0.001)]. Yellow has the greatest colour contrast to the fence background (e.g. trees or sky) across all light conditions, with all three test colours having similar levels of luminance contrast to the fence background. Furthermore, under shady conditions (often when the sun is behind the fence) current fence material (birch) has a higher luminance contrast to the background than all three of the test colours trialled. To an extent therefore, light conditions altered whether the three alternative colours tested had better, similar, or worse luminance contrast to the background than the traditional fence materials.

### 3.2 Behavioural responses to different fence colours

**Fig. 4:**
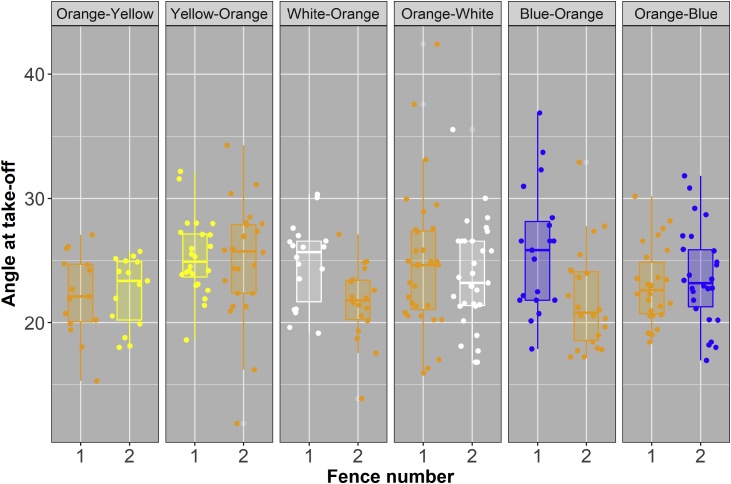
The angle of takeoff for horses jumping over fences of the control (orange) or test colour (white, fluorescent yellow, or bright blue) split by the trial pair sequence and whether the fence was the first (1) or second (2) fence that the horse jumped in the pair of test fences. Colour of box indicates fence colour.

**Fig. 5:**
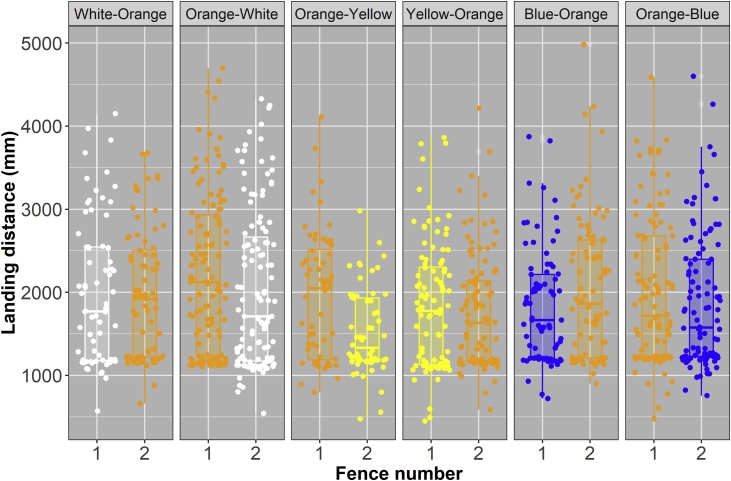
The landing distance (mm) of the front leading limb, front trailing limb, hind leading limb and hind trailing limb for horses jumping over fences of the control (orange) or test colour (white, fluorescent yellow, or bright blue) split by the trial pair sequence and whether the fence was the first (1) or second (2) fence that the horse jumped in the pair of test fences. Colour of box indicates fence colour.

Fence colour significantly affected the way a horse jumped the fence with regards to its takeoff and landing distances, and the angle of takeoff that a horse made during a jump. This effect varied depending on whether the colour (white, fluorescent yellow, or bright blue) was used on the first or second fence, and to an extent on whether it was the first, second, or third time that the horse was jumping the pair of fences (Table 5). Compared to orange, bright blue produced a significantly larger takeoff angle (Table 6) a difference that seems to have been be driven by the use of this colour on the first fence (Fig. 4). In terms of takeoff and landing distances, from the hind leading limb, horses jumping over white fences took off further away from the fence than when jumping over orange fences; that is they had a significantly larger takeoff distance from their hind leading limb (Table 6). There was no significant effect of fence colour on the takeoff distances for the other limbs (Table 5). Fence colour also had a significant effect on the landing distances of each limb (Table 5), this effect seems to have been predominantly driven by the effect of fluorescent yellow and bright blue fences, with horses landing closer to the fence when jumping over these fences than when jumping over an orange fence (Fig. 5; Table 6). It is worth noting however that the effect is much stronger for fluorescent yellow than bright blue fences (Table 6). Although colour significantly affected the total distance jumped by a horse (Table 5) there was no significant difference between the total distance jumped over the orange fence when compared to each of the three test fence colours (non-significant pairwise comparisons; Table 6).

**Table 5:** Results from mixed effects models testing each of the jumping parameters. The effect of fence colour on each of the different jumping parameters measured was tested using a linear mixed effects model, where each jumping parameter was a response variable; fence colour, fence sequence (the first or second fence in the pair of fences), and jump number (whether it was the 1^st^, 2^nd^, 3^rd^, or in rare cases 4^th^ time a horse had jumped the pair of fences) were fixed variables; and horse ID and trial day were crossed random effects.

Jump parameter	Fence colour				Fence number				Jump number			
**Angle at take-off**	**X^2^_1,3_ =**	10.61	**P =**	0.014	**X^2^_1,1_ =**	9.94	**P =**	0.002	**X^2^_1,3_ =**	10.59	**P =**	0.014
**Angle of bascule**	**X^2^_1,3_ =**	4.61	**P =**	0.203	**X^2^_1,1_ =**	0.42	**P =**	0.515	**X^2^_1,3_ =**	4.83	**P =**	0.185
**Height of wither over jump**	**X^2^_1,3_ =**	3.43	**P =**	0.330	**X^2^_1,1_ =**	1.97	**P =**	0.160	**X^2^_1,3_ =**	9.99	**P =**	0.019
**Total Jump Distance**	**X^2^_1,3_ =**	8.47	**P =**	0.037	**X^2^_1,1_ =**	0.45	**P =**	0.500	**X^2^_1,3_ =**	8.03	**P =**	0.045
**Breakdown of total jump distance components:**												
**Take-off distance – Distance from front leading limb and base of front of fence**	**X^2^_1,3_ =**	4.67	**P =**	0.198	**X^2^_1,1_ =**	3.20	**P =**	0.074	**X^2^_1,3_ =**	8.25	**P =**	0.041
**Take-off distance – Distance from front trailing limb and base of front of fence**	**X^2^_1,3_ =**	4.04	**P =**	0.258	**X^2^_1,1_ =**	2.46	**P =**	0.117	**X^2^_1,3_ =**	7.81	**P =**	0.050
**Take-off distance – Distance from hind leading limb and base of front of fence**	**X^2^_1,3_ =**	9.68	**P =**	0.021	**X^2^_1,1_ =**	2.18	**P =**	0.140	**X^2^_1,3_ =**	8.55	**P =**	0.036
**Take-off distance – Distance from hind trailing limb and base of front of fence**	**X^2^_1,3_ =**	7.07	**P =**	0.070	**X^2^_1,1_ =**	4.46	**P =**	0.035	**X^2^_1,3_ =**	9.58	**P =**	0.023
**Landing distance – Distance from front leading limb and base of rear of fence**	**X^2^_1,3_ =**	12.33	**P =**	0.006	**X^2^_1,1_ =**	18.70	**P <**	0.001	**X^2^_1,3_ =**	1.86	**P =**	0.601
**Landing distance – Distance from front trailing limb and base of rear of fence**	**X^2^_1,3_ =**	10.17	**P =**	0.017	**X^2^_1,1_ =**	16.25	**P <**	0.001	**X^2^_1,3_ =**	2.58	**P =**	0.460
**Landing distance – Distance from hind leading limb and base of rear of fence**	**X^2^_1,3_ =**	14.91	**P =**	0.002	**X^2^_1,1_ =**	3.82	**P =**	0.051	**X^2^_1,3_ =**	5.18	**P =**	0.159
**Landing distance – Distance from hind trailing limb and base of rear of fence**	**X^2^_1,3_ =**	10.94	**P =**	0.013	**X^2^_1,1_ =**	5.11	**P =**	0.024	**X^2^_1,3_ =**	6.36	**P =**	0.096

**Table 6:** Results of posthoc comparisons for those jumping parameters that were significantly affected by fence colour (see Table 5). Posthoc tests were carried out using the package = multcomp (Hothorn et al., 2008) to assess differences in the parameter of interest (e.g. Angle at take-off) between jumps made over orange fences and those made over fences of each of the three test colours (white, fluorescent yellow, or bright blue).

Jump parameter	Fence Pair	Estimate	+SE	z	P
**Angle at take-off**	Orange - White	−1.11	0.65	−1.70	0.246
	Orange - Fluoro Yellow	0.02	0.71	0.03	1.000
	Orange - Bright Blue	−2.00	0.68	−2.95	0.010
**Take-off distance – Distance from hind leading limb and base of front of fence**	Orange - White	−300.91	97.07	−3.10	0.006
	Orange - Fluoro Yellow	−112.29	105.41	−1.07	0.634
	Orange - Bright Blue	−22.91	100.64	−0.23	0.994
**Total Jump Distance**	Orange - White	−197.73	92.36	−2.14	0.094
	Orange - Fluoro Yellow	116.29	99.95	1.16	0.568
	Orange - Bright Blue	128.80	95.12	1.35	0.439
**Landing distance – Distance from front leading limb and base of rear of fence**	Orange - White	108.23	76.19	1.42	0.397
	Orange - Fluoro Yellow	203.77	83.27	2.45	0.043
	Orange - Bright Blue	177.56	78.50	2.26	0.069
**Landing distance – Distance from front trailing limb and base of rear of fence**	Orange - White	54.70	70.00	0.78	0.819
	Orange - Fluoro Yellow	175.44	75.88	2.31	0.061
	Orange - Bright Blue	161.47	71.70	2.25	0.071
**Landing distance – Distance from hind leading limb and base of rear of fence**	Orange - White	100.16	98.62	1.02	0.671
	Orange - Fluoro Yellow	339.78	107.83	3.15	0.005
	Orange - Bright Blue	232.27	102.50	2.27	0.069
**Landing distance – Distance from hind trailing limb and base of rear of fence**	Orange - White	121.55	89.81	1.35	0.439
	Orange - Fluoro Yellow	227.31	97.69	2.33	0.059
	Orange - Bright Blue	199.76	92.37	2.16	0.089

## 4 Discussion

The results show that current fence colouring, specifically the orange takeoff board and midrail, is not optimal for horse vision, and that weather and light conditions should be taken into account when considering alternative colours. To horse vision, the predicted contrast between the base of the fence (orange takeoff board) and the foreground, and the orange midrail with the mid-fence for the current fence colours and materials used, is poor. In most cases there was wide variation in conspicuousness of the top of fences against the background, most likely attributed to the highly variable nature of the background immediately adjacent to the top of the fence, i.e. sky/vegetation/stands. Blues, white, and yellows generally had much higher external and internal contrast than current fence material, but their suitability depends on the specific fence component in question. The colours that were most contrasting against the foreground, in comparison to the orange takeoff board, were blue and fluorescent yellow. Fluorescent yellow is also several times more contrasting across all fence backgrounds than natural brush and has considerably higher contrast to the main fence than the orange midrail, as does light green, likely due to this colour’s high luminance. Overall, the use of white, yellow, or blue would significantly improve the visibility of the takeoff board, midrail, and top of the fence/hurdle to horses. However, it is important to note that the exact shade, texture, and/or brightness properties of the white, yellow, or blue used influences the conspicuousness of these colours and that the suitability of each colour depends on the part of the fence in question. Light blues provide higher luminance contrast than darker blues, but blues and whites may blend in with the sky if used on the top of a fence with no treeline behind it. The choice of yellow is also key, as matt fluorescent yellow consistently has the highest colour and luminance contrast of all the colours tested, where as non-fluorescent shades are far less distinguishable from foreground turf, or other bright green vegetation. This is particularly important when considering the takeoff board, where a fluorescent yellow board would provide markedly improved contrast against the foreground, but a non-fluorescent shade of yellow would have similar contrast to both light green and the current orange colour used. Therefore, if fluorescent yellow cannot be sourced or is not financially viable to use on a large scale across an entire racecourse, white or light blue would be a more suitable alternative than non-fluorescent yellow.

There was a significant effect of weather/light conditions on the contrast of white, blue, yellow, and current fence components (takeoff board, midrail, and top edge of fence) to the foreground, main fence, and background. For each of the three fence edge comparisons (foreground vs. takeoff board, fence material vs. midrail, and fence edge vs. background) the colour contrast of the white, yellow, and blue was generally higher than the traditional fence colours, but this varied depending on the light conditions and the fence contrast in question, with shade significantly reducing the contrast under most scenarios. This was most true for the luminance JNDs for the foreground vs. takeoff board, to a lesser extent fence edge vs. background comparisons, but not the case for the midrail vs. fence material comparisons where the luminance contrast of the midrail and the three test colours did not vary according to light conditions. Interestingly, and potentially significantly, the contrast of blue and white versus the foreground was less affected by strong shadows than yellow, and strong shadows are most likely to arise at the base of a fence (such as when the sun is from behind). Blues and whites also had significantly lower chromatic contrast to the fence than the current orange midrail, although they had considerably higher luminance contrast, making yellow, with its consistently higher chromatic and achromatic contrast to fence material, overall the most conspicuous colour against all fence materials tested (i.e. birch, natural greenery, and artificial greenery).

For the behavioural trials, our experiment showed that the colour of the fences plays a role in both the shape that the horses made whilst jumping a fence and the total distance jumped. Horses jumping over fences with bright blue markers tended to have a larger angle at takeoff, compared to the orange fence, indicating that horses are jumping differently over these colours. Landing distances were significantly shorter when horses jumped over fences with fluorescent yellow markers and a similar, though non-significant, trend appeared to be driven by fences with bright blue markers. For both of these jumping parameters, effects were more pronounced when the bright blue or fluorescent yellow coloration was used in the first opposed to the second fence in the pair of fences. Lastly, horses jumping over fences with white markers had a larger takeoff distance, than when compared to the orange fence. Together these results indicate that horses jump differently depending on the colour of the fence, with differences between the control (orange) fence and each of the three test colours. There was also some deviation depending on how many times the horses jumped the fences (1–3), with the responses noted above weakening with an increasing number of jumps, but this effect was generally consistent across treatments and parameters.

Shorter landing distances (closer placement of limbs to the rear of the fence when landing) are often associated with greater jumping performance, whereas increased takeoff distance in some disciplines is linked to a lower likelihood of clearing an obstacle (Deuel and Park, 1991; Fercher, 2017; Wejer et al., 2013). Likewise, the angle at takeoff, represents the upwards trajectory of a jumping horse and is a key determinant of the nature of horse movement when clearing an obstacle, as well as its success in clearing that obstacle (Fercher, 2017; Powers and Harrison, 2000). In equine sports such as eventing, a larger angle of takeoff is sometimes linked to a higher or potentially a more rounded jump (Fercher, 2017). Although it is worth noting that one would therefore also expect jumps with larger angles at takeoff to have a larger clearing distance (height of withers over the jump) and a more rounded trunk at the midpoint of the jump (smaller angle of bascule), but this was not the case for the horses with larger takeoff angles in this study. One possible explanation for this disparity could be that the ideal angle at takeoff varies between different equine sports (de Godoi et al., 2014d; Lewczuk et al., 2006). In racing a flatter jump shape is generally favoured, compared to disciplines such as show jumping, as it maximises energy efficiency and reduces speed loss when clearing the jump. Different ‘jump shapes’ are also influenced by the training, as well as the breeds, used in particular equine sports, with individuals within these categories also often being acknowledged to have their own particular jumping ‘styles’ (Fercher, 2017; Wejer et al., 2013)

These results demonstrate that horses see and respond to the alternative fence colours chosen for the trial. The strength of the responses measured differed depending on the fence marked with the novel colour, i.e. whether fence 1 or fence 2 was marked with one of the three test colours, suggesting that there might have been a ‘fence order’ effect. This may have been related to colour novelty, although the fence number on which the novel colour (white, fluorescent yellow, or bright blue) was used first, differed between colour trials (e.g. in the fluorescent yellow trials, yellow was first used on fence 2 whereas in the white and bright blue trials it was used on fence 1). These differences may therefore be more likely attributable to a combination of the comparably longer lead in for the first fence or even horse and rider fatigue at the second fence. The latter may also have contributed to the decrease in the strength of the different jumping responses to each of the three test colours, over repeated jumps, although this could also reflect familiarisation of the horse with the alternative colours being used. Overall, the differing jumping response of horses in this experiment strongly suggests that horses see and respond to alterations in fence colouration. Fluorescent yellow and bright blue produced similar deviations in jumping parameters from orange, although bright blue alone caused changes in the angle at takeoff. Finally, these results should also be assessed through the lens of other sources of unavoidable potential variation associated with the study, such as differences in the jockeys and cohort of horses used in each trial, due availability constraints, and variation in the weather on trial days.

Our study shows that the current colours used as visibility features on fences and hurdles in UK horseracing are unlikely to be well designed to horse vision. In fact, several other colours would likely provide much greater visibility to horses and induce potentially beneficial behavioural responses. Nonetheless, there are other factors to consider besides direct visibility in the choice of obstacle colour. For example, lots of other features exist in the racecourse environment that are white (e.g. railings), meaning that white may potentially be confused with other objects in the visual scene. Otherwise, yellow is highly effective in all comparisons except under strong shadows, and these tend to occur at the base of fences, where yellow offers less of a visibility advantage over blue or white. Therefore, blue or white may be a better choice for features close to the ground. The downside of white, however, is that it may quickly become dirty, reducing its effect. As such, optimal fence design for horse vision may involve orange colours being replaced with a highly fluorescent white (or a light, highly luminant blue) for the takeoff board, and a fluorescent yellow for the midrail and for hurdles.

Ultimately, the work here requires testing in a standard racing environment before the full implications can be evaluated. This may include a range of courses and weather/light conditions. There is also a great deal of potential to further explore the role of colour and visibility in racing and training, including further analysis of performance across cohorts of horses and racing environments and the inclusion of more advanced biomechanical measurement techniques that can capture the forces and velocity involved in a jump (Clayton and Hobbs, 2017). Colour and visibility in the broader racing arena is likely to be important, including of non-fence colours and features around the courses (stands, vegetation, advertising boards), as seems to be the case in eventing (Stachurska et al., 2002). While humans are generally very good at seeing fluorescent yellow and white (hence the former’s use in high-visibility clothing), the visibility of different fence colours to jockeys during races and training should be considered too. Finally, our work here has also focussed on colour, yet horses have reduced ability to see fine detail and pattern to humans (*visual acuity*) (Timney and Keil, 1992), albeit with a visual streak across the retina of improved acuity (Harman et al., 1999). Horses also have marked differences in their level of binocular overlap to humans, and a blind spot in front of the head (Harman et al., 1999). These differences may have a similarly important effect on welfare and safety, and performance in training and racing as colour. Many other factors beyond the scope of our study here will likely also influence the responses of horses to fences, including cognition, long-term learning and prior experience, physiological state such as hormone levels, higher-level processing of colour and contrast, and beyond. Future work should investigate these and how they affect jump performance and responses to colour.

Our work here is directly relevant to other horse sports, such as eventing and show jumping, but also other areas such as greyhound racing, dog agility, and beyond, where colours and contrast may play an important role in responses and performance (Stachurska et al., 2010, 2002). More broadly, vision modelling and behavioural experiments are common place in studies of animal ecology and evolution (Renoult et al., 2017), yet rarely utilised in applied areas – there is great potential for these methods and approaches to help inform best practice in areas ranging from livestock welfare through to conservation in areas such as captive breeding and enrichment (Bizeray et al., 2002; Renoult et al., 2017).

## Funding

This work was supported by a research grant from the Racing Foundation and British Horseracing Authority, and an earlier Exeter internal impact accelerator funding and a BBSRC Pathfinder Award (BB/P017339/1), all to MS. Note that some of the funding for this research was provided by the British Horseracing Authority, who are responsible for the governance, administration and regulation of horseracing and the wider horseracing industry in Britain.
