# Supplementary material for: Horse vision and obstacle visibility in horseracing
Source: Appl Anim Behav Sci. 2020 Jan;222:104882. doi: 10.1016/j.applanim.2019.104882 (PMC6988441; doi:10.1016/j.applanim.2019.104882)
Supplement: Supplementary file 1 [file mmc1.docx]

**Horse vision and obstacle visibility in horseracing**

Sarah Catherine Paul & Martin Stevens

**Supplementary material 1 - Visibility of Different Fence/Hurdle Colours Under Different Light Conditions, Posthoc Test Results**

| **ColJND_diff** | **Estimate** | **Std. Error** | **z value** | **Pr(>\|z\|)** |
| --- | --- | --- | --- | --- |
| Takeoff_Board.OvercastDaytime - White.OvercastDaytime | -4.19 | 0.30 | -14.07 | <0.001 |
| Takeoff_Board.OvercastDaytime - Yellow.OvercastDaytime | -3.64 | 0.30 | -12.16 | <0.001 |
| Takeoff_Board.OvercastDaytime - Blue.OvercastDaytime | -5.08 | 0.30 | -17.00 | <0.001 |
| Takeoff_Board.OvercastEvening - White.OvercastEvening | -2.38 | 0.86 | -2.78 | 0.11 |
| Takeoff_Board.OvercastEvening - Yellow.OvercastEvening | -4.32 | 0.89 | -4.83 | <0.001 |
| Takeoff_Board.OvercastEvening - Blue.OvercastEvening | -3.58 | 0.85 | -4.19 | <0.001 |
| Takeoff_Board.ShadeDaytime - White.ShadeDaytime | -3.60 | 1.43 | -2.51 | 0.22 |
| Takeoff_Board.ShadeDaytime - Yellow.ShadeDaytime | 8.93 | 1.48 | 6.03 | <0.001 |
| Takeoff_Board.ShadeDaytime - Blue.ShadeDaytime | -4.47 | 1.43 | -3.12 | 0.038 |
| Takeoff_Board.ShadeEvening - White.ShadeEvening | -4.67 | 1.09 | -4.30 | <0.001 |
| Takeoff_Board.ShadeEvening - Yellow.ShadeEvening | 0.35 | 1.09 | 0.32 | 1.00 |
| Takeoff_Board.ShadeEvening - Blue.ShadeEvening | -5.50 | 1.09 | -5.06 | <0.001 |
| Takeoff_Board.Sunny_CloudCoverDaytime - White.Sunny_CloudCoverDaytime | -3.05 | 0.30 | -10.15 | <0.001 |
| Takeoff_Board.Sunny_CloudCoverDaytime - Yellow.Sunny_CloudCoverDaytime | -2.60 | 0.31 | -8.51 | <0.001 |
| Takeoff_Board.Sunny_CloudCoverDaytime - Blue.Sunny_CloudCoverDaytime | -3.90 | 0.30 | -12.98 | <0.001 |
| Takeoff_Board.Sunny_CloudCoverEvening - White.Sunny_CloudCoverEvening | -3.09 | 0.86 | -3.57 | 0.008 |
| Takeoff_Board.Sunny_CloudCoverEvening - Yellow.Sunny_CloudCoverEvening | -4.34 | 0.86 | -5.01 | <0.001 |
| Takeoff_Board.Sunny_CloudCoverEvening - Blue.Sunny_CloudCoverEvening | -3.86 | 0.86 | -4.47 | <0.001 |
| Takeoff_Board.SunnyDaytime - White.SunnyDaytime | -3.43 | 0.47 | -7.38 | <0.001 |
| Takeoff_Board.SunnyDaytime - Yellow.SunnyDaytime | -2.29 | 0.47 | -4.86 | <0.001 |
| Takeoff_Board.SunnyDaytime - Blue.SunnyDaytime | -4.28 | 0.47 | -9.18 | <0.001 |
| Takeoff_Board.SunnyEvening - White.SunnyEvening | -3.60 | 0.56 | -6.43 | <0.001 |
| Takeoff_Board.SunnyEvening - Yellow.SunnyEvening | -2.58 | 0.56 | -4.60 | <0.001 |
| Takeoff_Board.SunnyEvening - Blue.SunnyEvening | -4.43 | 0.56 | -7.90 | <0.001 |

Table 1. Post-hoc results for pairwise Tukey comparisons between the colour JND values for each of the test colours (white, yellow, and blue) and the takeoff board within each of the eight different light conditions. Comparisons run using model with factor representing combination of fence/colourboard and light conditions and (package: multcomp)

| **LumJND_diff** | **Estimate** | **Std. Error** | **z value** | **Pr(>\|z\|)** |
| --- | --- | --- | --- | --- |
| Takeoff_Board.OvercastDaytime - White.OvercastDaytime | -30.15 | 1.07 | -28.27 | <0.001 |
| Takeoff_Board.OvercastDaytime - Yellow.OvercastDaytime | -26.63 | 1.07 | -24.84 | <0.001 |
| Takeoff_Board.OvercastDaytime - Blue.OvercastDaytime | -23.75 | 1.07 | -22.24 | <0.001 |
| Takeoff_Board.OvercastEvening - White.OvercastEvening | -32.25 | 3.06 | -10.53 | <0.001 |
| Takeoff_Board.OvercastEvening - Yellow.OvercastEvening | -30.12 | 3.19 | -9.45 | <0.001 |
| Takeoff_Board.OvercastEvening - Blue.OvercastEvening | -29.19 | 3.04 | -9.60 | <0.001 |
| Takeoff_Board.ShadeDaytime - White.ShadeDaytime | 11.73 | 5.12 | 2.29 | 0.35 |
| Takeoff_Board.ShadeDaytime - Yellow.ShadeDaytime | 9.54 | 5.28 | 1.81 | 0.74 |
| Takeoff_Board.ShadeDaytime - Blue.ShadeDaytime | 15.26 | 5.12 | 2.98 | 0.06 |
| Takeoff_Board.ShadeEvening - White.ShadeEvening | -6.54 | 3.87 | -1.69 | 0.83 |
| Takeoff_Board.ShadeEvening - Yellow.ShadeEvening | -2.14 | 3.87 | -0.55 | 1.00 |
| Takeoff_Board.ShadeEvening - Blue.ShadeEvening | 0.57 | 3.87 | 0.15 | 1.00 |
| Takeoff_Board.Sunny_CloudCoverDaytime - White.Sunny_CloudCoverDaytime | -29.33 | 1.08 | -27.17 | <0.001 |
| Takeoff_Board.Sunny_CloudCoverDaytime - Yellow.Sunny_CloudCoverDaytime | -25.51 | 1.09 | -23.30 | <0.001 |
| Takeoff_Board.Sunny_CloudCoverDaytime - Blue.Sunny_CloudCoverDaytime | -22.93 | 1.08 | -21.24 | <0.001 |
| Takeoff_Board.Sunny_CloudCoverEvening - White.Sunny_CloudCoverEvening | -28.80 | 3.09 | -9.33 | <0.001 |
| Takeoff_Board.Sunny_CloudCoverEvening - Yellow.Sunny_CloudCoverEvening | -24.08 | 3.09 | -7.80 | <0.001 |
| Takeoff_Board.Sunny_CloudCoverEvening - Blue.Sunny_CloudCoverEvening | -22.80 | 3.09 | -7.39 | <0.001 |
| Takeoff_Board.SunnyDaytime - White.SunnyDaytime | -16.11 | 1.66 | -9.68 | <0.001 |
| Takeoff_Board.SunnyDaytime - Yellow.SunnyDaytime | -13.23 | 1.69 | -7.85 | <0.001 |
| Takeoff_Board.SunnyDaytime - Blue.SunnyDaytime | -10.91 | 1.67 | -6.53 | <0.001 |
| Takeoff_Board.SunnyEvening - White.SunnyEvening | -19.44 | 2.00 | -9.72 | <0.001 |
| Takeoff_Board.SunnyEvening - Yellow.SunnyEvening | -14.04 | 2.00 | -7.02 | <0.001 |
| Takeoff_Board.SunnyEvening - Blue.SunnyEvening | -14.32 | 2.00 | -7.16 | <0.001 |

Table 2. Post-hoc results for pairwise Tukey comparisons between the luminance JND values for each of the test colours (white, yellow, and blue) and the takeoff board within each of the eight different light conditions. Comparisons run using model with factor representing combination of fence/colourboard and light conditions and (package: multcomp)

| **ColJND_diff** | **Estimate** | **Std. Error** | **z value** | **Pr(>\|z\|)** |
| --- | --- | --- | --- | --- |
| Midrail.OvercastDaytime - White.OvercastDaytime | 0.84 | 0.06 | 14.36 | <0.001 |
| Midrail.OvercastDaytime - Yellow.OvercastDaytime | -1.79 | 0.06 | -30.54 | <0.001 |
| Midrail.OvercastDaytime - Blue.OvercastDaytime | 0.82 | 0.06 | 14.07 | <0.001 |
| Midrail.OvercastEvening - White.OvercastEvening | 1.19 | 0.19 | 6.42 | <0.001 |
| Midrail.OvercastEvening - Yellow.OvercastEvening | -1.39 | 0.19 | -7.42 | <0.001 |
| Midrail.OvercastEvening - Blue.OvercastEvening | 0.94 | 0.18 | 5.08 | <0.001 |
| Midrail.ShadeDaytime - White.ShadeDaytime | 1.07 | 0.18 | 5.93 | <0.001 |
| Midrail.ShadeDaytime - Yellow.ShadeDaytime | -1.93 | 0.18 | -10.49 | <0.001 |
| Midrail.ShadeDaytime - Blue.ShadeDaytime | 0.94 | 0.18 | 5.21 | <0.001 |
| Midrail.ShadeEvening - White.ShadeEvening | 0.12 | 0.24 | 0.49 | 1 |
| Midrail.ShadeEvening - Yellow.ShadeEvening | -2.47 | 0.24 | -10.33 | <0.001 |
| Midrail.ShadeEvening - Blue.ShadeEvening | -0.13 | 0.24 | -0.56 | 1 |
| Midrail.Sunny_CloudCoverDaytime - White.Sunny_CloudCoverDaytime | 1.01 | 0.06 | 16.48 | <0.001 |
| Midrail.Sunny_CloudCoverDaytime - Yellow.Sunny_CloudCoverDaytime | -1.84 | 0.06 | -29.59 | <0.001 |
| Midrail.Sunny_CloudCoverDaytime - Blue.Sunny_CloudCoverDaytime | 1.03 | 0.06 | 16.77 | <0.001 |
| Midrail.Sunny_CloudCoverEvening - White.Sunny_CloudCoverEvening | 0.90 | 0.14 | 6.24 | <0.001 |
| Midrail.Sunny_CloudCoverEvening - Yellow.Sunny_CloudCoverEvening | -2.04 | 0.14 | -14.12 | <0.001 |
| Midrail.Sunny_CloudCoverEvening - Blue.Sunny_CloudCoverEvening | 0.75 | 0.14 | 5.21 | <0.001 |
| Midrail.SunnyDaytime - White.SunnyDaytime | 1.27 | 0.10 | 13.30 | <0.001 |
| Midrail.SunnyDaytime - Yellow.SunnyDaytime | -1.73 | 0.10 | -17.94 | <0.001 |
| Midrail.SunnyDaytime - Blue.SunnyDaytime | 1.03 | 0.10 | 10.79 | <0.001 |
| Midrail.SunnyEvening - White.SunnyEvening | 0.66 | 0.12 | 5.41 | <0.001 |
| Midrail.SunnyEvening - Yellow.SunnyEvening | -1.70 | 0.12 | -13.91 | <0.001 |
| Midrail.SunnyEvening - Blue.SunnyEvening | 0.54 | 0.12 | 4.44 | <0.001 |

Table 3. Post-hoc results for pairwise Tukey comparisons between the colour JND values for each of the test colours (white, yellow, and blue) and the midrail within each of the eight different light conditions. Comparisons run using model with factor representing combination of fence/colourboard and light conditions and (package: multcomp)

| **LumJND_diff** | **Estimate** | **Std. Error** | **z value** | **Pr(>\|z\|)** |
| --- | --- | --- | --- | --- |
| Midrail - White | -31.50 | 0.66 | -48.03 | <0.001 |
| Midrail - Yellow | -28.34 | 0.66 | -43.00 | <0.001 |
| Midrail - Blue | -25.23 | 0.66 | -38.47 | <0.001 |

Table 4. Post-hoc results for pairwise Tukey comparisons between the luminance JND values for each of the test colours (white, yellow, and blue) and the midrail (package: multcomp), comparisons are across all light conditions as there was no significant interaction between midrail/test colour and light conditions (see main results).

| **ColJND_diff** | **Estimate** | **Std. Error** | **z value** | **Pr(>\|z\|)** |
| --- | --- | --- | --- | --- |
| Fence_Birch.OvercastDaytime - White.OvercastDaytime | 0.25 | 0.07 | 3.55 | 0.009 |
| Fence_Birch.OvercastDaytime - Yellow.OvercastDaytime | -1.97 | 0.07 | -28.11 | < 0.001 |
| Fence_Birch.OvercastDaytime - Blue.OvercastDaytime | -0.08 | 0.07 | -1.20 | 0.99 |
| Fence_Birch.OvercastEvening - White.OvercastEvening | 0.07 | 0.20 | 0.36 | 1.00 |
| Fence_Birch.OvercastEvening - Yellow.OvercastEvening | -2.23 | 0.20 | -10.94 | < 0.001 |
| Fence_Birch.OvercastEvening - Blue.OvercastEvening | -0.43 | 0.19 | -2.21 | 0.42 |
| Fence_Birch.ShadeDaytime - White.ShadeDaytime | -0.02 | 0.32 | -0.07 | 1.00 |
| Fence_Birch.ShadeDaytime - Yellow.ShadeDaytime | -1.41 | 0.33 | -4.20 | < 0.001 |
| Fence_Birch.ShadeDaytime - Blue.ShadeDaytime | -0.25 | 0.32 | -0.79 | 1.00 |
| Fence_Birch.ShadeEvening - White.ShadeEvening | -0.08 | 0.22 | -0.36 | 1.00 |
| Fence_Birch.ShadeEvening - Yellow.ShadeEvening | -2.58 | 0.22 | -11.65 | < 0.001 |
| Fence_Birch.ShadeEvening - Blue.ShadeEvening | -0.39 | 0.22 | -1.77 | 0.79 |
| Fence_Birch.Sunny_CloudCoverDaytime - White.Sunny_CloudCoverDaytime | 0.09 | 0.07 | 1.21 | 0.99 |
| Fence_Birch.Sunny_CloudCoverDaytime - Yellow.Sunny_CloudCoverDaytime | -2.02 | 0.07 | -27.30 | < 0.001 |
| Fence_Birch.Sunny_CloudCoverDaytime - Blue.Sunny_CloudCoverDaytime | -0.20 | 0.07 | -2.81 | 0.10 |
| Fence_Birch.Sunny_CloudCoverEvening - White.Sunny_CloudCoverEvening | 0.20 | 0.18 | 1.09 | 1.00 |
| Fence_Birch.Sunny_CloudCoverEvening - Yellow.Sunny_CloudCoverEvening | -2.07 | 0.18 | -11.48 | < 0.001 |
| Fence_Birch.Sunny_CloudCoverEvening - Blue.Sunny_CloudCoverEvening | -0.08 | 0.18 | -0.45 | 1.00 |
| Fence_Birch.SunnyDaytime - White.SunnyDaytime | -0.11 | 0.10 | -1.05 | 1.00 |
| Fence_Birch.SunnyDaytime - Yellow.SunnyDaytime | -2.31 | 0.10 | -22.27 | < 0.001 |
| Fence_Birch.SunnyDaytime - Blue.SunnyDaytime | -0.41 | 0.10 | -4.01 | 0.001 |
| Fence_Birch.SunnyEvening - White.SunnyEvening | 0.29 | 0.14 | 2.16 | 0.46 |
| Fence_Birch.SunnyEvening - Yellow.SunnyEvening | -1.94 | 0.14 | -14.27 | < 0.001 |
| Fence_Birch.SunnyEvening - Blue.SunnyEvening | 0.01 | 0.14 | 0.09 | 1.00 |

Table 5. Post-hoc results for pairwise Tukey comparisons between the colour JND values for each of the test colours (white, yellow, and blue) and the top edge of the fence within each of the eight different light conditions. Comparisons run using model with factor representing combination of fence/colourboard and light conditions and (package: multcomp)

| **LumJND_diff** | **Estimate** | **Std. Error** | **z value** | **Pr(>\|z\|)** |
| --- | --- | --- | --- | --- |
| Fence_Birch.OvercastDaytime - White.OvercastDaytime | 0.01 | 0.17 | 0.08 | 1.00 |
| Fence_Birch.OvercastDaytime - Yellow.OvercastDaytime | 0.37 | 0.17 | 2.18 | 0.45 |
| Fence_Birch.OvercastDaytime - Blue.OvercastDaytime | 0.63 | 0.17 | 3.74 | 0.00 |
| Fence_Birch.OvercastEvening - White.OvercastEvening | -1.24 | 0.47 | -2.64 | 0.16 |
| Fence_Birch.OvercastEvening - Yellow.OvercastEvening | -1.06 | 0.49 | -2.16 | 0.47 |
| Fence_Birch.OvercastEvening - Blue.OvercastEvening | -0.97 | 0.47 | -2.09 | 0.52 |
| Fence_Birch.ShadeDaytime - White.ShadeDaytime | 3.58 | 0.77 | 4.63 | <0.001 |
| Fence_Birch.ShadeDaytime - Yellow.ShadeDaytime | 3.76 | 0.81 | 4.66 | <0.001 |
| Fence_Birch.ShadeDaytime - Blue.ShadeDaytime | 4.38 | 0.77 | 5.67 | <0.001 |
| Fence_Birch.ShadeEvening - White.ShadeEvening | 3.57 | 0.53 | 6.70 | <0.001 |
| Fence_Birch.ShadeEvening - Yellow.ShadeEvening | 3.81 | 0.53 | 7.15 | <0.001 |
| Fence_Birch.ShadeEvening - Blue.ShadeEvening | 4.60 | 0.53 | 8.62 | <0.001 |
| Fence_Birch.Sunny_CloudCoverDaytime - White.Sunny_CloudCoverDaytime | -1.46 | 0.17 | -8.48 | <0.001 |
| Fence_Birch.Sunny_CloudCoverDaytime - Yellow.Sunny_CloudCoverDaytime | -1.14 | 0.18 | -6.38 | <0.001 |
| Fence_Birch.Sunny_CloudCoverDaytime - Blue.Sunny_CloudCoverDaytime | -0.90 | 0.17 | -5.24 | <0.001 |
| Fence_Birch.Sunny_CloudCoverEvening - White.Sunny_CloudCoverEvening | -0.01 | 0.43 | -0.01 | 1.00 |
| Fence_Birch.Sunny_CloudCoverEvening - Yellow.Sunny_CloudCoverEvening | 0.23 | 0.43 | 0.53 | 1.00 |
| Fence_Birch.Sunny_CloudCoverEvening - Blue.Sunny_CloudCoverEvening | 0.21 | 0.43 | 0.50 | 1.00 |
| Fence_Birch.SunnyDaytime - White.SunnyDaytime | -2.02 | 0.25 | -8.18 | <0.001 |
| Fence_Birch.SunnyDaytime - Yellow.SunnyDaytime | -1.65 | 0.25 | -6.60 | <0.001 |
| Fence_Birch.SunnyDaytime - Blue.SunnyDaytime | -1.50 | 0.25 | -6.06 | <0.001 |
| Fence_Birch.SunnyEvening - White.SunnyEvening | 0.17 | 0.33 | 0.52 | 1.00 |
| Fence_Birch.SunnyEvening - Yellow.SunnyEvening | 0.51 | 0.33 | 1.54 | 0.92 |
| Fence_Birch.SunnyEvening - Blue.SunnyEvening | 0.52 | 0.33 | 1.59 | 0.89 |

Table 6. Post-hoc results for pairwise Tukey comparisons between the luminance JND values for each of the test colours (white, yellow, and blue) and the top edge of the fence within each of the eight different light conditions. Comparisons run using model with factor representing combination of fence/colourboard and light conditions and (package: multcomp)
